# Nieuwe wegen naar arbocuratieve samenwerking: samen werken van praktijkondersteuners huis- en bedrijfsarts bij multiproblematiek

**DOI:** 10.1007/s12508-022-00374-7

**Published:** 2022-11-30

**Authors:** Emma Vossen, Frederieke G. Schaafsma, Joost W. J. van der Gulden, Cornelis A. de Kock, Rosanne Schaap, Johannes R. Anema, Joost A. G. M. van Genabeek

**Affiliations:** 1 Afdeling Public and Occupational Health, Amsterdam Public Health Research Institute, Amsterdam UMC, Amsterdam, Nederland; 2 Afdeling Eerstelijnsgeneeskunde, Radboudumc, Nijmegen, Nederland; 3 Huisartsenpraktijk de Bie en de Kock, Deurne, Nederland; 4 TNO Work Health Technology, Leiden, Nederland; 5 HAN University of Applied Sciences, Nijmegen, Nederland

**Keywords:** arbocuratieve samenwerking, praktijkondersteuner, eerstelijnsgezondheidszorg, bedrijfsgezondheidszorg, multiproblematiek, Interprofessional cooperation, Assistant practitioner, Primary health care, Occupational health care, Practice nurse

## Abstract

*Achtergrond: *Momenteel is er nauwelijks sprake van arbocuratieve samenwerking tussen de eerstelijns- en bedrijfsgezondheidszorg. Waar eerdere initiatieven tot verbetering vooral gericht waren op de huis- en bedrijfsarts, onderzoeken we in deze bijdrage welke rol praktijkondersteuners van de huisarts (POH-ggz en POH-S) en van de bedrijfsarts (POB) voor zichzelf zien bij multiproblematiek. Tevens exploreren we welke belemmeringen er zijn voor arbocuratieve samenwerking door praktijkondersteuners bij multiproblematiek.

*Methode: *We hebben drie focusgroepgesprekken uitgevoerd met zeven POH’s-ggz, elf POH’s‑S en acht POB’s – 26 praktijkondersteuners in totaal.

*Resultaten: *De praktijkondersteuners in ons onderzoek komen tijdens hun werk in aanraking met multiproblematiek. POH’s en POB’s zien een rol voor zichzelf weggelegd bij het bespreken en het bieden van ondersteuning bij respectievelijk werk- en privégerelateerde klachten. Daarbij erkennen ze, waar nodig, het belang van arbocuratieve samenwerking om goede zorg te leveren. Op dit moment vindt er echter geen directe samenwerking plaats op het niveau van de praktijkondersteuner. Belemmeringen hiervoor blijken de formele regels rond taakdelegatie en rolopvatting van de POB, onbekendheid en vooroordelen bij vooral POH’s wat betreft de bedrijfsgezondheidszorg, en praktische barrières als de AVG-wetgeving en bereikbaarheid.

*Conclusie: *POH’s en POB’s staan open voor arbocuratieve samenwerking, mits een oplossing gevonden wordt voor deze fundamentele en praktische belemmeringen.

## Abstract (en)

*Background: *To this date, there is little to no interprofessional collaboration between professionals in general and occupational health care in the Netherlands. Where earlier initiatives for improvement focused on general practitioners (GPs) and occupational physicians (OPs), we examine the role that professionals working under task delegation of GPs and OPs can play in addressing problems on multiple life domains as well as in interprofessional collaboration.

*Methods: *We conducted three focus group interviews with 7 assistant practitioners (APs) in general practice, 11 practice nurses (PNs) in general practice and 8 APs in occupational health practice.

*Results: *All PNs and APs in our study are confronted with multi-domain problems and see a role for themselves in addressing these problems. Moreover, in case of multi-domain problems, they acknowledge the relevance of interprofessional collaboration to provide good care. At this moment, however, there is practically no collaboration at the level of PNs and APs. Challenges are found in the formal task delegation and role identity of the APs in occupational health practice, unfamiliarity and prejudices among especially general practice PNs and APs regarding occupational health care, and practical barriers as privacy laws and reachability.

*Conclusion: *Interprofessional collaboration among PNs and APs in general and occupational health care is possible, provided that a solution is found for fundamental and practical challenges. Potential solutions are to shift the focus from sharing medical information to communicating about the needs of working patients to function healthily on multiple life domains, to address interprofessional collaboration in educations, to adapt consultation protocols to include work and to organize joint meetings between professionals in general and occupational health practice. Finally, addressing structural barriers such as privacy laws and financing requires political action.

## Inleiding

Al decennia wordt geprobeerd de samenwerking tussen de bedrijfsgezondheidszorg en curatieve zorg (‘arbocuratieve samenwerking’) te bevorderen. De eerste initiatieven waren gericht op het stimuleren van samenwerking tussen de huis- en bedrijfsarts via communicatieformulieren en -protocollen [1]. Later is in meerdere projecten geëxperimenteerd met het onderbrengen van de bedrijfsarts in de huisartsenpraktijk [2–4]. Het idee was dat het bij elkaar brengen van de verschillende professionals zou leiden tot betere arbocuratieve samenwerking, en daarmee tot het voorkomen van ziekteverzuim, en het bevorderen van werkhervatting en de gezonde inzetbaarheid in werk van patiënten. Hoewel de betrokken artsen arbocuratieve samenwerking tijdens de diverse projecten als positief en zinvol ervoeren, stopte deze samenwerking doorgaans zodra het project eindigde. Redenen hiervoor waren dat faciliteiten vanuit de projecten wegvielen, zoals directe beschikbaarheid van een bedrijfsarts in de huisartsenpraktijk, en de projectduur te kort was om daadwerkelijke veranderingen tot stand te brengen [3, 4]. Vaak genoemde belemmeringen voor structurele samenwerking zijn gebrek aan tijd en financiering, maar ook vooroordelen over en onbekendheid met elkaars werk [5–8]. Na al deze pogingen is het de vraag hoe deze belemmeringen op een duurzame manier geslecht kunnen worden.

Door de scheiding tussen behandeling en werkgerelateerde zorg in Nederland is er in de eerstelijnsgezondheidszorg van oudsher beperkt aandacht voor werk [5, 9, 10]. Tegelijkertijd ervaart naar schatting een derde van de werkende patiënten die de huisarts bezoekt (tevens) werkgerelateerde klachten en verwachten patiënten zelf dat ook huisartsen aandacht besteden aan de relatie tussen gezondheid en werk [10, 11]. Aandacht voor werk binnen de eerstelijnsgezondheidszorg is daarnaast extra relevant voor de grote groep werkenden die geen toegang heeft tot de bedrijfsgezondheidszorg, zoals zzp’ers [5, 10]. Onderzoek heeft laten zien dat huisartsen door het stellen van vijf korte vragen, werkgerelateerde klachten vroegtijdig kunnen signaleren, vaak nog voordat er sprake is van verzuim [12]. In de bedrijfsgezondheidszorg is niet goed onderzocht welk percentage werknemers verzuimt door privégerelateerde problematiek. Bedrijfsartsen vragen privégerelateerde problemen wel uit, maar er wordt weinig gedaan om deze problematiek aan te pakken [13]. Hoewel huis- en bedrijfsartsen dus te maken krijgen met problematiek uit respectievelijk de bedrijfs- en de eerstelijnsgezondheidszorg, kan er een verbeterslag gemaakt worden als het gaat om (1) het bespreken en aanpakken van problemen op meerdere levensgebieden (‘multiproblematiek’) en (2) het waar nodig aangaan van arbocuratieve samenwerking. Met multiproblematiek bedoelen we in dit onderzoek ‘problemen op meerdere levensgebieden, zoals […] een combinatie van gezondheidsproblemen en problemen op het terrein van bijvoorbeeld sociale contacten, opvoeding en ontwikkeling, financiën, arbeid of wonen’ [14].

De laatste jaren zijn diverse nieuwe functies ontstaan waarin, afhankelijk van de opleidingsachtergrond, gedelegeerde taken van de huis- of bedrijfsarts overgenomen worden [15, 16]. Binnen de huisartsenpraktijk zijn dat de praktijkondersteuners somatiek (POH-S) en geestelijke gezondheidszorg (POH-ggz), de physician assistant (PA), verpleegkundig specialist (VS) en de praktijkverpleegkundige huisartsenzorg (PVH). In de bedrijfsgezondheidszorg is onlangs de functie van praktijkondersteuner bedrijfsgezondheidszorg (POB) geïntroduceerd, naast de al langer bestaande arboverpleegkundige, casemanager en verzuimconsulent. Het besteden van aandacht aan werk binnen de eerstelijnszorg en arbocuratieve samenwerking zouden onderdeel kunnen uitmaken van de gedelegeerde taken. Zo wordt op basis van een recent onderzoek naar werkgerelateerde zorg in de eerste lijn geadviseerd om deze zorg te beleggen bij POH’s, omdat werk bij zowel chronische aandoeningen (POH-S), als psychische problematiek (POH-ggz) vaak een rol speelt [9].

Het doel van het huidige onderzoek is te exploreren welke rol zowel POH’s als POB’s voor zichzelf zien in de behandeling van multiproblematiek en in het bijzonder van respectievelijk werk- en privégerelateerde klachten. Tevens willen we achterhalen wat hun ervaringen zijn met arbocuratieve samenwerking. Hiermee hopen we nieuwe wegen te vinden voor samenwerking tussen de eerstelijns- en de bedrijfsgezondheidszorg.

## Methoden

In dit onderzoek is gebruikgemaakt van de focusgroepmethode [17]. Op deze manier konden we via interactie tussen de deelnemende POH’s en POB’s, rolopvattingen en ervaringen rond multiproblematiek en arbocuratieve samenwerking exploreren onder praktijkondersteuners.

Dit onderzoek is goedgekeurd door de Medisch-Ethische Toetsingscommissie van het VUmc, onder nummer 2021.0332.

### Werving en onderzoeksgroep

In 2021 zijn drie focusgroepen gehouden met 26 praktijkondersteuners, respectievelijk met zeven POH’s-ggz, elf POH’s‑S en acht POB’s. Deelnemers zijn geworven via de klankbordgroep van het overkoepelende onderzoek waartoe dit deelonderzoek behoort [18]. Afgevaardigden van een zorgaanbieder en twee opleidingsinstituten uit de klankbordgroep hebben naar aanleiding van onze oproep een bijeenkomst gepland tijdens hun tweewekelijkse intervisiebijeenkomst (POH’s-ggz) of tijdens een gastcollege aan het eind van de opleiding (POH’s‑S en POB’s). Bij deze laatste gaat het om post-hbo-opleidingen voor verpleegkundigen (POH-S) en andere professionals met een hbo-opleiding (POB), die bij aanvang minimaal acht uur per week werkzaam zijn binnen een huisartsenpraktijk of onder begeleiding van een bedrijfsarts. De deelnemende praktijkondersteuners zijn daarmee allemaal werkzaam in deze functie en dus bekend met de praktijk. Deelnemers waren overwegend vrouwen (23), hetgeen in ieder geval representatief is voor de beroepsgroep van POH’s (volgens het NIVEL was in 2016 96% van de POH’s vrouw [19]); voor de vrij nieuwe functie van de POB’s is dit nog niet onderzocht. Vanwege de inpassing van de focusgroepen in bestaande bijeenkomsten hebben we de verschillende praktijkondersteuners in aparte groepen gesproken.

### Gegevensverzameling en -analyse

Alle focusgroepen hadden dezelfde opzet: na een introductie en kennismakingsronde volgde een korte uitleg over de achtergrond van het onderzoek. Vervolgens vond het groepsinterview plaats, gestructureerd aan de hand van een casus van een fictieve, mannelijke automonteur van 52 jaar met multiproblematiek, die een hartinfarct heeft gehad en na een geleidelijke re-integratie sinds vier maanden weer volledig aan het werk is. Deze werkende patiënt komt op controle bij de POH‑S, is doorverwezen naar de POH-ggz of verschijnt op het spreekuur van de POB vanwege zorgen over een hoge bloeddruk, problemen in de privésituatie en hoge werkdruk. Zie kader 1 voor de casusbeschrijving. De casus werd besproken aan de hand van drie thema’s waaraan vragen gekoppeld waren over: (1) herkenning van multiproblematiek tijdens consulten, (2) de rol van de praktijkondersteuner bij multiproblematiek en (3) ervaringen met arbocuratieve samenwerking. Alle focusgroepen werden begeleid door auteurs EV en FS. De bijeenkomsten duurden anderhalf uur. Vanwege de COVID-19-pandemie vonden de bijeenkomsten online plaats, via MS Teams of Zoom.

De focusgroepgesprekken zijn met toestemming van de deelnemers opgenomen en getranscribeerd, waarbij alle deelnemers geanonimiseerd zijn. De transcripten zijn in een eerste stap van open coderen [20] door auteur EV gelezen en van trefwoorden voorzien met behulp van het softwareprogramma MAXQDA. Auteur RS heeft het transcript van één focusgroepgesprek open gecodeerd, waarna EV en RS de trefwoorden hebben vergeleken en bediscussieerd om tot overeenstemming te komen. Vervolgens zijn deze trefwoorden tijdens het axiale coderen door EV geclusterd naar meer abstracte begrippen, op basis van de drie hoofdthema’s. De resultaten zijn ook per groep (POH-ggz, POH‑S, POB) vergeleken om onderlinge verschillen en overeenkomsten te achterhalen. Selectief coderen is niet toegepast omdat theorieontwikkeling geen doel was van dit onderzoek.

#### Kader 1. Ondersteun Peter bij een beter leven!

Peter is onderhoudsmonteur (52 jaar) en heeft een jaar geleden een hartinfarct gehad. Hij heeft al jaren een hoge bloeddruk en denkt dat werkdruk mede veroorzaker is van het hartinfarct. Na een geleidelijke re-integratie is hij sinds vier maanden weer volledig aan het werk.

(POH-S: Voor een controle-afspraak komt Peter bij je in de praktijk.) (POH-ggz: Peter is door zijn huisarts naar jou als POH-ggz doorverwezen. Voor een gesprek komt Peter bij je in de praktijk.) (POB: Peter komt bij jou op het preventief spreekuur voor een consult.) Peter geeft tijdens het consult aan dat hij zich zorgen maakt over hoe lang hij dit werk nog vol kan houden. Hij heeft het gevoel dat hij weer in zijn oude werkpatroon terugvalt en dat daarmee ook de werkdruk toeneemt. Hierdoor komt hij vaak oververmoeid thuis. Thuis heeft hij ook nauwelijks rustmomenten. Daarnaast maakt hij zich veel zorgen over zijn gezondheid. Zijn bloeddruk is nu onder controle, maar door de hoge werkdruk en zijn thuissituatie is Peter bang dat hij weer last krijgt van een hoge bloeddruk.

## Resultaten

Hieronder bespreken we de resultaten van de focusgroepen aan de hand van de drie thema’s (1) herkenning van multiproblematiek tijdens consulten, (2) de rol van de praktijkondersteuner bij multiproblematiek en (3) ervaringen met arbocuratieve samenwerking.

### Herkenning van multiproblematiek

Zowel POH’s als POB’s beseffen dat er bij de patiënten of werknemers die op hun consult komen soms problemen spelen op meerdere levensgebieden. Dit komt volgens hen vaker voor als er sprake is van een lagere sociaaleconomische status (SES). Het gaat dan om een combinatie van problemen, zoals gezondheidsklachten, werk en financiën, maar ook sociale of relatieproblematiek en problemen rond kinderen of mantelzorg. Volgens de POH’s en POB’s is multiproblematiek complex omdat het ene probleem het andere probleem in de hand werkt of versterkt. Een POB noemt het ‘lastig dat je niet altijd invloed hebt op alle factoren waar men tegenaan loopt’. Een POH-ggz en een POH‑S geven aan dat de cliënt bij multiproblematiek vaak het overzicht mist en dat het daarom belangrijk is om alle problemen in kaart te brengen om hulp te kunnen bieden:

‘Het overzicht hebben ze […] vaak niet, ze weten eigenlijk ook niet precies waardoor ze ziek zijn geworden. Ze kunnen heel veel dingen opnoemen, maar wat nou de directe aanleiding is geweest is heel ingewikkeld en die is ook niet te vinden. Dus de oplossing is daardoor ook niet zo heel makkelijk hè? Het creëren van overzicht en het geven van uitleg, dat […] vind ik vaak de belangrijkste dingen in zo’n gesprek. Dat je kijkt van, wat is er nou allemaal aan de hand?’ (POH-ggz)

‘Dan is het een optelsom van problemen op het werk, onhandig gedrag, […] stresseten en ontregeling en daarmee is de cirkel rond. Dus dan kan ik wel gaan trekken aan die diabetes, maar er is meer aan de hand. En het één voedt het ander en het is dan zo’n vicieuze cirkel die de verkeerde kant op wil.’ (POH-S)

### De rol van de praktijkondersteuner bij multiproblematiek

Tabel 1 toont de antwoorden op de vraag naar de rol van de praktijkondersteuner bij multiproblematiek. Zowel POH’s als POB’s zien voor zichzelf een rol weggelegd bij het uitvragen van multiproblematiek, om de verschillende oorzaken van gezondheidsklachten of verzuim te achterhalen. Hieronder valt ook het bespreken van werkgerelateerde problematiek door POH’s en niet-werkgerelateerde problematiek door POB’s. Volgens de praktijkondersteuners krijgt de patiënt of werknemer hierdoor overzicht in (oorzaken van) zijn of haar problematiek, wat het herstel of de re-integratie kan bevorderen.

Als volgende stap in het aanpakken van multiproblematiek zien de POH’s en POB’s het als hun taak om te kijken waar zijzelf en waar anderen ondersteuning kunnen bieden, zodat de patiënt of werknemer voldoende ondersteund wordt om weer in balans te komen. Hierin ervaren de POH’s‑S en POB’s wel beperkingen in hun rol: enerzijds door een tekort aan consulttijd, anderzijds door hun beperkte invloed op problemen op andere levensgebieden (zie tab. 1). Deze beperkingen bij het aanpakken van multiproblematiek, in het bijzonder van werkgerelateerde problemen, worden door de POH’s-ggz niet genoemd.

**Table 1:** 

rol van de praktijkondersteuner	uitwerking	citaten (selectie)
rol bij multiproblematiek	rol van de POH-ggz	‘Dat is altijd wat ik vraag, ja: waar bestaat je dag uit, wat doe je? Werk, ontspanning, vrije tijd, eten, slapen, relaties, sociaal netwerk, dat zijn standaardvragen en wat ik in kaart wil hebben.’ [fgPOHGGZ_r346]
	rol van de POH‑S	‘Ik denk dat dat er heel erg aan ligt, wat de patiënt zelf al gaat vertellen, als die meteen heel veel vertelt dan hoef je niet veel uit te vragen, maar als je het er echt uit moet trekken, dan zou ik wel wat meer op de domeinen ingaan.’ [fgPOHS_r509]
	rol van de POB	‘Het punt is meestal dat je wel goed doorvraagt en het goed in kaart probeert te brengen, dus eigenlijk kijken welke kern of kernen kunnen we vinden van de problematiek.’ [fgPOB_r331]
rol bij (niet-)werkgerelateerde problematiek	rol POH-ggz bij werk	‘Als POH-ggz vinden wij werk heel belangrijk, wij zeggen: het biedt afleiding en structuur aan de dag. Soms heb je als POH-ggz ook echt wel belang dat iemand teruggaat naar werk.’ [fgPOHGGZ_r550]
	rol POH‑s bij werk	‘Dat laatste [arbocuratieve samenwerking] zie ik denk ik tenminste op ons niveau nog niet gebeuren, maar het eerste wel: het sterker maken van die werknemer in de hoop dat het bespreekbaar gemaakt wordt daar waar het in ieder geval óók hoort [op het werk].’ [fgPOHS_r395]
	rol POB buiten werk	‘Je probeert zo breed mogelijk uit te vragen over BRAVO, de leefstijl, hoe dat iemand er thuis in zit.’ [fgPOB_r356]
beperkingen aan de eigen rol bij multiproblematiek	beperkingen aan rol van de POH‑S	‘Natuurlijk probeer je alles wel mee te pakken, je probeert een luisterend oor te bieden, maar uiteindelijk ben je daar wel om natuurlijk die […] diabetes gewoon zo goed mogelijk in te stellen.’ [fgPOHS_r40]
	beperkingen aan rol van de POB	‘Ik vraag wel door, maar je kunt sommige dingen ook […] weet je, ergens houdt het op wat wij kunnen betekenen.’ [fgPOB_r30]

*fg* focusgroep, *r* regelnummer in transcript waar het citaat te vinden is

### Ervaringen met arbocuratieve samenwerking

Zoals geïllustreerd in tab. 2, herkennen zowel POH’s als POB’s het belang van arbocuratieve samenwerking: door samenwerking kunnen zij betere zorg leveren en tegenstrijdige adviezen voorkomen. Toch is er geen sprake van arbocuratieve samenwerking. Sommige POH’s en POB’s sturen de patiënt of werkende door naar respectievelijk de bedrijfsarts of de huisarts. Anderen delen informatie per brief, vaak via de huis- of bedrijfsarts, of ze vragen gespreksverslagen op bij de patiënt met als doel ze samen door te spreken. POH’s en POB’s hebben echter geen direct fysiek of telefonisch overleg met professionals in respectievelijk de bedrijfs- en eerstelijnsgezondheidszorg.

**Table 2:** 

belang van samenwerking	citaten (selectie)
POH-ggz	Het is eigenlijk obligaat dat het zinvol is, ik bedoel, de werkomstandigheden en hoe de patiënt daarmee omgaat en hoe je iemand daarin dan kan begeleiden, als je daar goed overleg over hebt dat dat dan positief werkt. Ik bedoel, in feite heb je allemaal het belang van die patiënt voor ogen […]. Het wordt vaak door de patiënt als tegenstrijdig ervaren en ik denk dat als je goed overlegt, dat je kunt laten zien dat dat niet zo is. fgPOHGGZ_r657
POH‑S	Ik denk dat we samen toch hetzelfde doel hebben, dat we de patiënt gewoon […], ja in al zijn domeinen om het zo maar te zeggen, dat hij daar goed kan functioneren, of zo, zo goed mogelijk met de chronische ziekte die hij heeft, want dat zijn de mensen die we zien. Die chronische ziekte zal niet weggaan en hoe gaat hij daarmee om in al zijn domeinen, dus, ja, dan kunnen we voor elkaar wel wat betekenen. fgPOS_r813
POB	Ik vind dat inderdaad echt iets heel bijzonders om eigenlijk daar [bij problematiek buiten het werk] ook samenwerking in op te zoeken, want dat het werk daar ook een hele goede positieve rol in kan betekenen voor wat betreft wat iemand wél op de rails houdt, om te kijken van: wat heeft iemand nodig, sociale steun, afleiding, structuur. fgPOB_r104

*fg* focusgroep, *r* regelnummer in transcript waar het citaat te vinden is

Waarom is er op dit moment geen sprake van samenwerking? Tabel 3 laat zien met welke uitdagingen POH’s en POB’s geconfronteerd worden om de samenwerking aan te gaan. Deze uitdagingen blijken, naast praktische barrières als bereikbaarheid en AVG-wetgeving, fundamenteel van aard te zijn: ze raken de kern van de beroepen. Dat geldt vooral voor de rol- en taakomschrijving van de POB. Zo vormt de formele taakdelegatie tussen bedrijfsarts en POB een belemmering voor rechtstreekse arbocuratieve samenwerking voor POB’s. In de Werkwijzer Taakdelegatie staat beschreven dat ‘informatie opvragen bij de behandelaar, opstellen machtiging en vragen’ een taak is die niet door de bedrijfsarts mag worden gedelegeerd [21]. De POB’s in de focusgroepen zagen, in lijn met de Werkwijzer Taakdelegatie, het aangaan van samenwerking niet als onderdeel van hun rol. Ze geven als uitleg hiervoor dat ze geen (para)medische achtergrond hebben (geen BIG-registratie) en hun rol dan ook niet definiëren als behandelaar of zorgverlener, maar meer als casemanager van het re-integratieproces. In die hoedanigheid geven ze aan vaker contact te hebben met werkgevers (leidinggevenden, human resource managers) over werkgerelateerde problematiek van werknemers, dan met zorgprofessionals. In hun huidige rol ervaren ze zelden reden om met de laatstgenoemden contact te zoeken. De huidige rolopvatting van de POB wordt dus ervaren als een barrière voor arbocuratieve samenwerking.

Vooral voor de POH’s zijn vooroordelen over en onbekendheid met de bedrijfsgezondheidszorg barrières voor het aangaan van arbocuratieve samenwerking. In de focusgroepen gaven ze aan niet bekend te zijn met de collega’s van de bedrijfsgezondheidszorg en ook niet goed te weten wat hun taken en verantwoordelijkheden zijn. ‘Onbekend maakt onbemind’ werd vaker genoemd als reden voor het gebrek aan samenwerking door de POH’s, wat ook herkend werd door de POB’s. Het is voor de POH’s niet duidelijk wat er bijvoorbeeld met de opgevraagde informatie wordt gedaan. Sommigen maken zich zorgen, vooral over de (on)partijdigheid van de bedrijfsarts. De POH’s-ggz gaven daarbij aan dat ze ervaren dat bedrijfsartsen hen niet altijd serieus nemen en dat deze ook niet weten welke rol de POH-ggz precies speelt.

Ten slotte vertelden zowel POH’s als POB’s dat arbocuratieve samenwerking bemoeilijkt wordt door praktische barrières als onderlinge onbereikbaarheid en de AVG-wetgeving, waardoor eerst toestemming van de patiënt of werknemer gevraagd moet worden voor het uitwisselen van informatie. Deze barrières verhogen de drempel om de samenwerking op te zoeken.

**Table 3:** 

uitdagingen voor samenwerking	nadere uitleg	citaten (selectie)
werkwijzer taakdelegatie	arbocuratieve samenwerking is een taak van de bedrijfsarts en niet van de POB	‘Ik moet wel zeggen dat de afstemming vaak wel echt iets is voor de bedrijfsarts.’ [fgPOB_r458] ‘Het is afhankelijk van de afspraken die je hebt met de bedrijfsarts met wie je samenwerkt. Ik heb bijvoorbeeld met mijn bedrijfsarts de afspraak: het overleg met de curatieve sector doet hij en het overleg met de providerboog, dus die vanuit de werkgever wordt aangeboden, dat doe ik.’ [fgPOB_r475] ‘[Mijn arbodienst] heeft daar een heel duidelijke mening over, […] dat de bedrijfsarts de aanvraag doet voor de opvraag van medische gegevens.’ [fgPOB_r505]
rolopvatting van de POB	POB hebben geen BIG-registratie	‘Ik heb geen medische opleiding, dus mijn bedrijfsarts en ik hebben gewoon die keuze gemaakt: de curatieve sector doet hij en wat ik doe is dan vaak veel meer gericht op het werk.’ [fgPOB_r479] ‘De meesten die hier zitten hebben een niet-medische achtergrond […], we zijn niet BIG-geregistreerd. Daar hangt wel vanaf in hoeverre de bedrijfsarts jou mogelijkheden biedt om te overleggen.’ [fgPOB_r491]
	POB is geen behandelaar of zorgverlener	‘De POH is iets vaker behandelaar. Wij zijn geen behandelaars […]. Wij zijn, wij doen taken, echt taken gedelegeerd van een bedrijfsarts, dat is wat anders.’ [fgPOB_r528] ‘We zijn geen, in die zin, verlengstuk van de bedrijfsarts, maar onze rol moet je veel meer schatten richting een organisatie op organisatiekant. Veel meer op een stukje werkhervatting, re-integratie, hoe ga je om met opstartadviezen, de link naar de leidinggevende toe.’ [fgPOB_r532]
onderlinge vooroordelen en onbekendheid	wantrouwen bij POH’s richting de bedrijfsarts vanuit (eigen) ervaringen	‘Er zijn zo ongelooflijk veel bedrijfsartsen en […] met de één zal je een goede ervaring hebben en met de ander een slechte, maar wat ik nu in de nierzorg heb ervaren is dat mijn ervaringen vooral niet zo positief waren.’ [fgPOHS_r234] ‘Mijn ervaring is dat er veel wantrouwen is naar het bedrijf […]. Ze hebben zoiets van: het zijn twee handen op één buik, dat gevoel heb ik. Sommige bedrijfsartsen gedragen zich ook als zodanig.’ [fgPOHGGZ_r105]
	onbekendheid bij POH’s met bedrijfsarts	‘Dat gevoel dat de mensen soms hebben dat een bedrijfsarts onder één hoedje speelt met de werkgever, is dat ook zo, of is een bedrijfsarts, moet dat eigenlijk wel een goede vertrouwenspersoon zijn?’ [fgPOHS_r423] ‘Door alles heen klinkt voor mij toch nog te veel de rode lijn: onbekend is onbemind.’ [fgPOHS_r773] ‘Daar schort, denk ik, ook iets aan onze kant aan, dat het niet duidelijk genoeg is bij dit opvragen van de informatie wat er dan dus inderdaad de bedoeling mee is.’ [fgPOB_r263]
	onbekendheid met POH-ggz	‘Soms ben ik met iemand in gesprek en dan heeft iemand bij een bedrijfsarts te horen gekregen ja, maar een POH-ggz is niet voldoende, dat moet een psycholoog zijn.’ [fgPOHGGZ_r559]
praktische barrières	bereikbaarheid	‘Het is eigenlijk in de praktijk amper op elkaar afgestemd dat jij op het moment dat je die patiënt in de kamer hebt, contact hebt met de bedrijfsarts.’ [fgPOHGGZ_r590]
	AVG-wetgeving	‘In het kader van de AVG mogen ze dat niet zomaar delen, telefonisch, dus dan moet er eerst toestemming zijn [van de patiënt]. Ik bedoel, het wordt heel complex gemaakt ook.’ [fgPOB_295]

*fg* focusgroep, *r* regelnummer in transcript waar het citaat te vinden is

## Beschouwing

De POH’s en POB’s in ons onderzoek werken op dit moment niet samen met collega’s uit respectievelijk de bedrijfs- of eerstelijnsgezondheidszorg als er sprake is van multiproblematiek. Hoewel zowel POH’s als POB’s openstaan voor meer samenwerking, zijn belemmeringen terug te voeren op de vraag of ze deze samenwerking wel *kunnen* aangaan (onderlinge bereikbaarheid, AVG-wetgeving), *willen* aangaan (als gevolg van vooroordelen en onbekendheid bij vooral POH’s) en – meer fundamenteel – of ze de samenwerking wel *mogen* (taakdelegatie tussen bedrijfsarts en POB) en *moeten* aangaan (hoort deze bij de rol van de POB?). Waar de eerste twee afwegingen ook werden gezien in eerder onderzoek onder huis- en bedrijfsartsen [6, 7], raken de afwegingen rond de formele taakdelegatie en rolopvatting de kern van de functie van POB’s.

### Sterke en zwakke punten

Voor zover wij weten is de rol van praktijkondersteuners op dit gebied niet eerder onderzocht. Via focusgroepen is geëxploreerd welke rol POH’s‑S, POH’s-ggz en POB’s voor zichzelf zien bij het behandelen van multiproblematiek en bij arbocuratieve samenwerking. Hiermee werden deze thema’s vanuit meerdere perspectieven belicht. Een eventuele beperking is dat de deelnemende POH’s‑S en POB’s aan het eind van hun opleiding zaten, waardoor hun antwoorden mogelijk niet het handelen en denken van de gehele (meer ervaren) beroepsgroep weerspiegelen. Hoewel dit bij de POB’s niet naar voren kwam, vonden enkele POH’s‑S het door een gebrek aan ervaring lastig om sommige vragen – vooral die over het omgaan met multiproblematiek – te beantwoorden. Ze houden zich tijdens de consulten nog vooral bezig met het eigen taakgebied en hebben daardoor minder oog en tijd voor andere thema’s. Mogelijk voelen meer ervaren POH’s‑S zich meer toegerust om binnen de consulttijd ook aandacht te besteden aan problemen op het gebied van werk. Een tweede mogelijke beperking is dat de drie leden van de klankbordgroep die de focusgroep organiseerden en deelnemers wierven een sterke mening hebben over arbocuratieve samenwerking en daarmee minder representatief zijn. Hoewel dit zal gelden voor de drie klankbordleden, zal hier in mindere mate sprake van zijn bij de deelnemers, die vanuit hun opleiding (POH‑S en POB) of vanuit hun betrokkenheid bij de intervisiegroep (POH-ggz) (verplicht) deelnamen.

### Implicaties voor arbocuratieve samenwerking

In ons onderzoek bleken de Werkwijzer Taakdelegatie en het ontbreken van een BIG-registratie belemmeringen voor arbocuratieve samenwerking. Een mogelijke oplossing om tot meer arbocuratieve samenwerking tussen POH’s en POB’s te komen, is door het gesprek uit de medische hoek te halen en in plaats daarvan een mogelijkheid te bieden om laagdrempelig te communiceren met een collega uit het andere zorgdomein. Hoewel de cocreatie van oplossingen niet het doel was van het onderhavige onderzoek, werd tijdens de focusgroepbijeenkomsten gesuggereerd dat overleg tussen praktijkondersteuners niet hoeft te gaan over medische feiten, maar kan dienen ‘om gewoon je zorgen uit te spreken over wat je gehoord hebt en waarvan je vindt dat er iets mee moet gebeuren’. Volgens deze opvatting is arbocuratieve samenwerking niet beperkt tot (alleen) het delen van medische informatie, maar is het ook een manier om te kijken wat de werkende patiënt nodig heeft om op meerdere levensgebieden gezond te functioneren. Dit sluit goed aan bij ontwikkelingen waarbij gezondheid breder wordt gezien dan een medisch te duiden probleem, zoals bij de theorie over Positieve Gezondheid [22]. Bovendien past deze oplossing bij ontwikkelingen om niet langer alleen naar de gezondheidsbeperkingen van de werkende patiënt te kijken, maar ook naar diens mogelijkheden, zoals binnen de Positieve Psychologie gedaan wordt [23, 24]. Voorwaarde is dan wel dat bestaande protocollen en voorschriften rond taakdelegatie binnen zowel de eerstelijns- als de bedrijfsgezondheidszorg (zoals de Werkwijzer Taakdelegatie) deze manier van samenwerken mogelijk maken.

Onbekendheid met elkaars werk en zeker vooroordelen zijn hardnekkige belemmeringen voor arbocuratieve samenwerking, wat ook in onderzoek onder huis- en bedrijfsartsen naar voren kwam [6, 7]. Recentelijk werd door de Kwaliteitstafel Bedrijfs- en Verzekeringsgeneeskunde nog het advies gegeven om te blijven investeren in een imagocampagne om het spreekwoordelijke ‘onbekend maakt onbemind’ ongeldig te maken, zowel binnen de geneeskunde als daarbuiten [25]. Een mogelijkheid om deze belemmeringen aan te pakken is om al in de opleidingen tot POH-S/ggz en POB aandacht aan arbocuratieve samenwerking en het domein van respectievelijk de bedrijfs- en eerstelijnsgezondheidszorg te besteden. Dat gebeurt nu nog weinig. Tegelijkertijd kan interesse onder POH’s‑S en POH’s-ggz voor het thema ‘werk’ en arbocuratieve samenwerking voor huisartsen aanleiding zijn om de taakdelegatie naar POH’s binnen de huisartsenpraktijk te verruimen. In hun consulten zouden POH’s standaard kunnen vragen naar eventuele problemen op het werk in relatie tot de gezondheidsproblematiek van de patiënt. Tot slot kunnen wederzijdse onbekendheid en vooroordelen aangepakt worden door het stimuleren van gezamenlijke bijeenkomsten, zoals conferenties en symposia, of door regionale of lokale overlegvormen waarin zowel POH’s als POB’s vertegenwoordigd zijn.

Een andere belemmering voor arbocuratieve samenwerking tussen de eerstelijns- en de bedrijfsgezondheidszorg heeft betrekking op het ontbreken van structurele financiering om deze samenwerking aan te gaan [6–8, 25, 26]. Deze belemmering laat zich niet zo gemakkelijk oplossen, omdat de eerstelijns- en de bedrijfsgezondheidszorg in Nederland gescheiden bekostigingsstelsels kennen. Hierdoor zijn er aan beide kanten onvoldoende prikkels om te investeren op de snijvlakken van de eerstelijns- en de bedrijfsgezondheidszorg [26]. Zo beschouwen de Nederlandse overheid en zorgverzekeraars werkgerelateerde zorg primair als een taak van de bedrijfsgezondheidszorg, die bekostigd wordt door werkgevers [9]. Investeringen van zorgverzekeraars in aandacht voor werk door POH’s en arbocuratieve samenwerking zouden financiële baten kunnen opleveren voor werkgevers en uitvoeringsorganisaties van sociale zekerheid (UWV en gemeenten), zonder dat deze partijen bijdragen in de kosten [9]. In de bedrijfsgezondheidszorg lijken verzuimverzekeraars nog weinig op preventie gericht en bieden ook de contracten van werkgevers met arbodiensten en bedrijfsartsen weinig ruimte voor preventieve activiteiten [5, 9, 25, 26]. Arbocuratieve samenwerking met de eerstelijnszorg biedt ook hier blijkbaar nog geen kloppend verdienmodel [9]. Om arbocuratieve samenwerking te stimuleren in beide zorgdomeinen blijft het daarom belangrijk om ook op hoger niveau na te denken over mogelijkheden voor een andere financieringsstructuur.

Ten slotte is nader onderzoek nodig naar de manier waarop huisartsen en bedrijfsartsen aankijken tegen arbocuratieve samenwerking tussen POH’s en POB’s. Naast de vraag of en welke zorgprofessionals willen samenwerken, is het ook relevant om te onderzoeken of werkende patiënten behoefte hebben aan deze samenwerking. Tot zover is er weinig onderzoek gedaan vanuit het patiëntperspectief, vooral bij werkenden met een lagere SES [27–29]. Eerder onderzoek laat zien dat patiënten hier welwillend tegenover staan, mits zij toestemming hebben gegeven en ze zelf de regie kunnen houden over de informatie-uitwisseling [28, 29]. Vervolgonderzoek kan bijvoorbeeld gericht zijn op de vraag of patiënten samenwerking tussen praktijkondersteuners zouden waarderen als het gaat om werken met chronische aandoeningen of psychische problematiek.

## Conclusie

Dit onderzoek laat zien dat praktijkondersteuners binnen de eerstelijns- en bedrijfsgezondheidszorg niet of nauwelijks samenwerken, ook niet als er sprake is van multiproblematiek. Hoewel zij hier wel voor openstaan, zijn er naast belemmeringen als de AVG-wetgeving en bereikbaarheid, ook meer fundamentele belemmeringen (regels over taakdelegatie, rolopvattingen, onbekendheid en vooroordelen over elkaars werk), die moeten worden geslecht wil men POH’s en POB’s stimuleren tot meer samenwerking en afstemming in het belang van de werkende patiënt. Mogelijke oplossingen zijn het uit de medische hoek halen van samenwerking op het niveau van de POH en POB, het besteden van aandacht aan arbocuratieve samenwerking en de bedrijfs- of eerstelijnsgeneeskunde in de opleiding tot POH-S/ggz en POB, het aanpassen van consultprotocollen of het organiseren van gezamenlijke bijeenkomsten voor POH’s en POB’s. Voor het oplossen van structurele belemmeringen, zoals de AVG-wetgeving en financiering, is op politiek niveau overleg nodig.
